# Lipid Metabolism-Related Gene Signature Predicts Prognosis and Indicates Immune Microenvironment Infiltration in Advanced Gastric Cancer

**DOI:** 10.1155/2024/6639205

**Published:** 2024-02-26

**Authors:** Lijian He, Qiange Ye, Yanmei Zhu, Wenqi Zhong, Guifang Xu, Lei Wang, Zhangding Wang, Xiaoping Zou

**Affiliations:** ^1^Department of Gastroenterology, Nanjing Drum Tower Hospital, School of Medicine, Jiangsu University, Nanjing, Jiangsu Province, China; ^2^Department of Gastroenterology, Tongling People's Hospital, Tongling, Anhui Province, China; ^3^Department of Gastroenterology, Nanjing Drum Tower Hospital Clinical College of Nanjing University of Chinese Medicine, Nanjing, Jiangsu Province, China; ^4^Department of Gastroenterology, Nanjing Drum Tower Hospital, The Affiliated Hospital of Nanjing University Medical, Nanjing, Jiangsu Province, China; ^5^Department of Gastroenterology, Affiliated Taikang Xianlin Drum Tower Hospital, Medical School of Nanjing University, Nanjing, Jiangsu Province, China

## Abstract

**Objective:**

Abnormal lipid metabolism is known to influence the malignant behavior of gastric cancer. However, the underlying mechanism remains elusive. In this study, we comprehensively analyzed the biological significance of genes involved in lipid metabolism in advanced gastric cancer (AGC).

**Methods:**

We obtained gene expression profiles from The Cancer Genome Atlas (TCGA) database for early and advanced gastric cancer samples and performed differential expression analysis to identify specific lipid metabolism-related genes in AGC. We then used consensus cluster analysis to classify AGC patients into molecular subtypes based on lipid metabolism and constructed a diagnostic model using least absolute shrinkage and selection operator- (LASSO-) Cox regression analysis and Gene Set Enrichment Analysis (GSEA). We evaluated the discriminative ability and clinical significance of the model using the Kaplan-Meier (KM) curve, ROC curve, DCA curve, and nomogram. We also estimated immune levels based on immune microenvironment expression, immune checkpoints, and immune cell infiltration and obtained hub genes by weighted gene co-expression network analysis (WGCNA) of differential genes from the two molecular subtypes.

**Results:**

We identified 6 lipid metabolism genes that were associated with the prognosis of AGC and used consistent clustering to classify AGC patients into two subgroups with significantly different overall survival and immune microenvironment. Our risk model successfully classified patients in the training and validation sets into high-risk and low-risk groups. The high-risk score predicted poor prognosis and indicated low degree of immune infiltration. Subgroup analysis showed that the risk model was an independent predictor of prognosis in AGC. Furthermore, our results indicated that most chemotherapeutic agents are more effective for AGC patients in the low-risk group than in the high-risk group, and risk scores for AGC are strongly correlated with drug sensitivity. Finally, we performed qRT-PCR experiments to verify the relevant results.

**Conclusion:**

Our findings suggest that lipid metabolism-related genes play an important role in predicting the prognosis of AGC and regulating immune invasion. These results have important implications for the development of targeted therapies for AGC patients.

## 1. Introduction

Recently, gastric cancer (GC) has become the fifth most commonly diagnosed cancer and the fourth leading cause of cancer-related deaths worldwide [1]. According to the pathological changes, GC can be classified into early gastric cancer (EGC) and advanced gastric cancer (AGC). EGC is characterized by tumor lesions confined to the mucosa or submucosa, while AGC involves carcinoma infiltration beyond the submucosa into the muscular layer or beyond [2]. EGC and AGC have different prognoses, with a five-year survival rate of over 95% for EGC and only about 30% for AGC [3, 4]. Despite the application of newer treatment modalities, including surgery, chemotherapy, and immunotherapy to AGC, its prognosis remains unsatisfactory [5]. One of the major reasons for the poor prognosis of AGC is its high propensity for metastasis [6]. Thus, identifying a new signature for AGC prognosis is crucial.

Tumor cells require more energy for survival and proliferation than normal cells [7]. Metabolic reprogramming is one of the important phenotypes of tumor cells and is increasingly appreciated. Metabolic reprogramming is an important phenotype of tumor cells that is increasingly recognized. Recent studies suggest that lipid metabolism is closely associated with the rapid proliferation, survival, migration, invasion, and metastasis of malignant tumors [8]. Numerous investigations have highlighted a significant elevation in lipid content in both plasma and tissues among individuals with gastric cancer compared to their normal counterparts [9, 10]. Furthermore, the association between mutations or abnormal expression of specific lipid metabolism-related genes and gastric cancer occurrence has been established. Notably, signaling molecules such as sterol regulatory element-binding proteins 1 and 2 (SREBP1 and SREBP2) play crucial roles in lipid metabolism regulation [11, 12]. Sterol O-acyltransferase 1 (SOAT1) has been identified as a promoter of lymph node metastasis in gastric cancer through the activation of the SREBP pathway [13]. CD36, known for its involvement in fatty acid uptake, has been implicated in promoting gastric cancer metastasis [14]. Moreover, disruptions in lipid metabolism have implications for the therapeutic landscape and prognosis of gastric cancer. Existing research has demonstrated the influence of lipid metabolism disorders on chemotherapy and radiotherapy efficacy, closely intertwining them with gastric cancer prognosis [15]. Noteworthy examples include statins, which exhibit the potential to prolong survival postsurgery and adjuvant chemotherapy in gastric cancer patients [16]. Additionally, ATP-binding cassette subfamily G member 2 (ABCG2) has been identified as a contributor to increased resistance to cisplatin treatment in gastric cancer patients. Furthermore, serum cholesterol levels have emerged as a prognostic indicator for gastric cancer patients [17].

The tumor microenvironment (TME) comprises tumor cells, immune cells, extracellular matrix, and mesenchymal tissue [18]. The tumor immune microenvironment (TIME) is a component of the TME composed of different immune cells that can play a key role in cancer development, progression, and control by engaging with the solid part of the tumor [19]. Immune cells, such as T cells and bone marrow mesenchymal stem cells, have been shown to further promote gastric carcinogenesis based on H. pylori infection [20, 21]. Immune cells in the immune microenvironment also play an important role in the inflammatory cancer transformation of the stomach [22]. The impact of lipid reprogramming on the immune microenvironment of tumors is complex. Lipid metabolism is integral for the survival and proliferation of both immune cells and tumor cells, playing a pivotal role in the regulation of tumor cell processes such as proliferation, invasion, and metastasis. Furthermore, it holds significant implications for the activation, differentiation, and function of immune cells within the tumor microenvironment [23]. In this intricate milieu, lipid metabolism emerges as a critical determinant influencing the activity, proliferation, and differentiation of immune cells, thereby shaping their ability to target tumor cells effectively [24]. Additionally, the modulation of oxidative stress and metabolic substance concentrations within the tumor microenvironment by lipid metabolism can impact tumor cell immune evasion and drug resistance [25, 26]. For example, fatty acid oxidation (FAO) can enhance the efficiency of PD-1 immunotherapy [27], and excessive intake of fatty acids may affect NK cells, which are originally powerful antitumor fighters, or even promote immune escape [28]. The impact of the immune microenvironment on cancer is unquestionable. Exploring the relationship between TIME and AGC will help deepen our understanding of this disease.

In this study, we aimed to investigate the impact of lipid metabolism-related gene (LMRG) expression levels on the prognosis of AGC patients. Using differential gene expression analysis, we identified two groups with differential lipid metabolism expression and constructed a risk model for LMRG that can serve as an indicator of AGC prognosis. Furthermore, we identified a close association between LMRG and TIME in AGC. Our study sheds light on the role of LMAG in AGC and provides information that may help clinicians improve patient prognosis and develop personalized treatment plans for AGC.

## 2. Materials and Methods

### 2.1. Data Source and Preprocessing

We obtained high-throughput sequencing gene expression data and clinical information of stomach adenocarcinoma (STAD) patients from TCGA database, including 374 STAD tissues and 27 normal tissues. The mRNA sequencing data was transformed to transcript per kilobase million (TPM) values followed by log2(*x* + 1) transformation, which is considered the most accurate quantification method with minimal statistical bias. Samples with missing clinical factors or survival follow-up information were excluded. The GSE62254 dataset, containing 300 STAD samples, was downloaded from the NCBI Gene Expression Omnibus (GEO) database and used as a validation set to assess the accuracy of the prognostic features. Clinical information of STAD patients was extracted from TCGA database, and after removing samples with unclear clinical information, patients were classified into early stage (T1, 19 cases) and advanced stage (T2-4, 347 cases) according to the pathological TNM criteria.

### 2.2. Identification of Differentially Expressed Genes (DEGs)

The limma package of R was used to analyze the differential gene expression between EGC and AGC tissues, using a significance threshold of *p*.adj < 0.05 and log2 fold change (|log2FC|) ≥ 1.5. The differential gene expression results were visualized using ggplot2, pheatmap, and volcano plots.

### 2.3. Lipid Metabolism-Related Gene Collection

We extracted 743 genes considered to be related to lipid metabolism from the “METABOLISM_OF_LIPIDS” gene set in MSigDB (http://www.broadinstitute.org/gsea/msigdb/, M24779) for further analysis.

#### 2.3.1. Consensus Cluster Analysis

Consensus clustering is an unsupervised classification technique implemented through multiple resampling and clustering. We used six genes related to lipid metabolism with prognostic value to classify patients using “ConsensusClusterPlus” with KM clustering using 1-Pearson correlation distance and 2000 replicate resamplings of 80% of the samples. The appropriate number of clusters (*k*) was determined based on the cumulative distribution function (CDF) plot. The stability of the clusters was further validated in the principal component analysis (PCA).

#### 2.3.2. Construction of the Protein-Protein Interaction (PPI) Network

The PPI network was constructed using the STRING database to analyze the interactions between genes or proteins. The obtained PPI information was visualized using Cytoscape software.

### 2.4. Weighted Gene Coexpression Analysis (WGCNA)

WGCNA was used to construct an unsigned coexpression network to identify coexpression modules. First, we identified DEGs between the two clusters using the “limma” package and calculated the median absolute deviation (MAD) of the DEGs. The top 50% of genes with the smallest MAD were excluded, and the outlier genes and samples were removed by the good sample gene method of the R package WGCNA. The soft threshold power (*β*) was estimated to construct the biologically important scale-free network. The topological overlap matrix (TOM) was calculated based on the adjacency matrix, and the dynamic tree cutting algorithm was used to detect gene modules. We calculated gene significance (GS), module membership (MM), and related modules with clinical features, which represent the relationship between gene expression profiles and module signature genes. Gene salience represents the absolute value of the association between gene expression and module traits.

### 2.5. Functional Enrichment Analysis

Kyoto Encyclopedia of Genes and Genomes (KEGG) functional enrichment analysis was performed using the “clusterProfiler” software package, with a significance threshold of *p* < 0.05. Biological functions of different subtypes of AGC were evaluated based on cell composition (CC), molecular function (MF), biological process (BP), biological pathways, diseases, and drugs.

### 2.6. Gene Set Enrichment Analysis (GSEA)

GSEA was conducted using the “clusterProfiler” package to identify significant differences in gene sets between different clusters in the MSigDB set (c2.cp.v7.2.symbols.gmt) enrichment set. The significance threshold was set to *p* < 0.05 and FDR < 0.25.

### 2.7. Tumor Immune Microenvironment Analysis

The ESTIMATE and CIBERSORT algorithms were used to calculate immune infiltration scores for each sample using RNA-seq data. Differences in immune infiltration between different tumor subtypes were evaluated using a *t*-test statistical method with a significance level of *p* < 0.05. The expression of key immune checkpoint genes, including PDCD1, LAG3, CTLA4, CD274 (PD-L1), and BTLA, was also investigated.

### 2.8. Development and Validation of Risk Models

A valid prediction model was developed using LASSO-Cox analysis. The most useful predictive features were derived from the training cohort using overall survival (OS), and a risk score was calculated for each patient in both the training and validation cohorts. The X-Tile software was used to calculate the optimal cut-off value to divide patients into high-risk and low-risk groups. Kaplan-Meier survival curves were used to determine the model ability to distinguish different subtypes of patients, and time-dependent receiver operating characteristic (ROC) curves and decision curve analysis (DCA) curves were used to assess the efficiency.

### 2.9. Evaluation of Drug Sensitivity

The “oncoPredict” package was used to calculate the IC_50_ values of AGC after multidrug treatment, and the person correlation test was used to compare the differences in IC_50_ between the commonly used antitumor drugs in the high-risk and low-risk groups. Box plots were generated using the R package “ggplot2.”

### 2.10. Single-Cell Analysis

The Single-Cell Center for Tumor Immunity (TISCH) was used to visualize the expression level of lipid metabolism-related genes at the level of individual cells using the STAD_GSE134520 dataset.

### 2.11. Patients and Specimens

A total of 25 GC tissues, which were pathologically confirmed advanced-stage GC, along with the corresponding adjacent noncancerous fresh frozen tissues, were collected from patients who underwent radical gastrectomy at the Nanjing Drum Tower Hospital. Subsequently, these tissues were utilized for further qRT-PCR analysis. All participants granted informed consent, in accordance with the guidelines and approval of the Ethics Committee of Nanjing Drum Tower Hospital (no. 2020-103).

### 2.12. Validation of the Expression Levels of Critical Genes by qRT-PCR

Total RNA was extracted from human and mouse GC tissues, as well as corresponding noncancerous tissues, using TRIzol reagent (Invitrogen, Carlsbad, CA, USA) following the manufacturer's protocol. Subsequent reverse transcription (RT) was executed with the Reverse Transcription Kit (Vazyme, Nanjing, China). RT-PCR reactions were carried out using the SYBR Green PCR Kit (Vazyme, Nanjing, China), conducted in triplicate, and analyzed on an Applied Biosystems 7900HT sequence detection system (Applied Biosystems). The thermal cycling parameters were as follows: initial enzyme activation and denaturation were conducted for 10 minutes at 95°C, following by 40 amplification cycles, each comprising denaturation at 95°C for 30 seconds, annealing at 60°C for 60 seconds, and elongation at 72°C for 60 seconds. Finally, a dissociation curve stage was implemented with intervals of 60 seconds at 95°C, 30 seconds at 55°C, and 30 seconds at 95°C. GAPDH served as the internal control for mRNA normalization. Relative expression levels of AKR1B1, OSBPL1A, PRKD1, ABCA1, CD36, and FABP3 were determined using the comparative 2−*ΔΔ*Ct method. Detailed primer sequences utilized are provided in Table 1.

### 2.13. Statistical Analysis

Data normalization and statistical analysis were performed using R software (version 4.2.1) and related packages. For *p* value calculations, a two-tailed paired *t*-test was utilized for within-group comparisons, and a two-sample *t*-test was applied for between-group analyses. For comparisons involving more than two groups, a one-way ANOVA test followed by Tukey's multiple comparisons was conducted. The Cox regression analysis was used to identify independent prognostic factors, and ROC curves were used to assess the accuracy of risk characteristics. KM curves and the logarithmic rank test were used to analyze differences in overall survival. All statistical tests were two sided, and *p* < 0.05 was considered to be significant.

## 3. Results

### 3.1. Identification of Differentially Expressed Lipid Metabolism-Related Genes in STAD and PPI Network Construction

**Figure 1:**
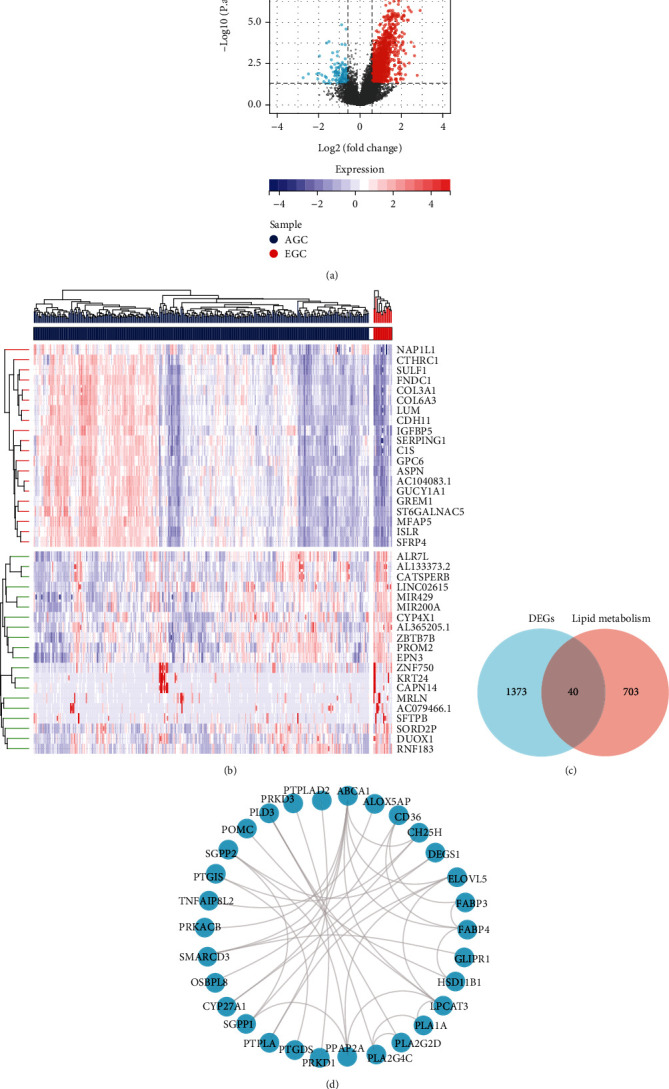
Identification of lipid metabolism-related differential genes in STAD and construction of PPI networks. (a) Volcanic maps of AGC and EGC, with upregulated genes in red and downregulated genes in blue. (b) Heat maps of the 20 upregulated and 20 downregulated genes with the most significant differences. (c) The intersection of differential genes and lipid metabolism-related genes was used to obtain 40 lipid metabolism-related differential genes. (d) PPI network was constructed with 40 differential genes related to lipid metabolism.

We obtained 374 gastric cancer samples from TCGA website and performed differential analysis on AGC (T2-4) and EGC (T1) using the limma software package after excluding samples with incomplete clinical information. Volcano and heat maps were generated to visualize the differential expression of genes (Figures 1(a) and 1(b)). A total of 1413 DEGs were identified using the criterion that the absolute log fold change (logFC) was greater than 0.585 (1.5-fold change) and the adjusted *p* value < 0.05, including 1297 upregulated genes and 116 downregulated genes. The heat map highlighted the top 20 upregulated and 20 downregulated genes (Figure 1(b)). We also retrieved 743 LMRGs from the GSEA database and identified 40 overlapping genes between DEGs and LMRGs (Figure 1(c)). To explore the relationships among these 40 genes, we constructed a PPI network using the STRING database with a medium correlation degree threshold (0.4) and visualized it using Cytoscape 3.6.1 software (Figure 1(d)).

### 3.2. Prognostic Screening of Lipid Metabolism-Related Genes

**Figure 2:**
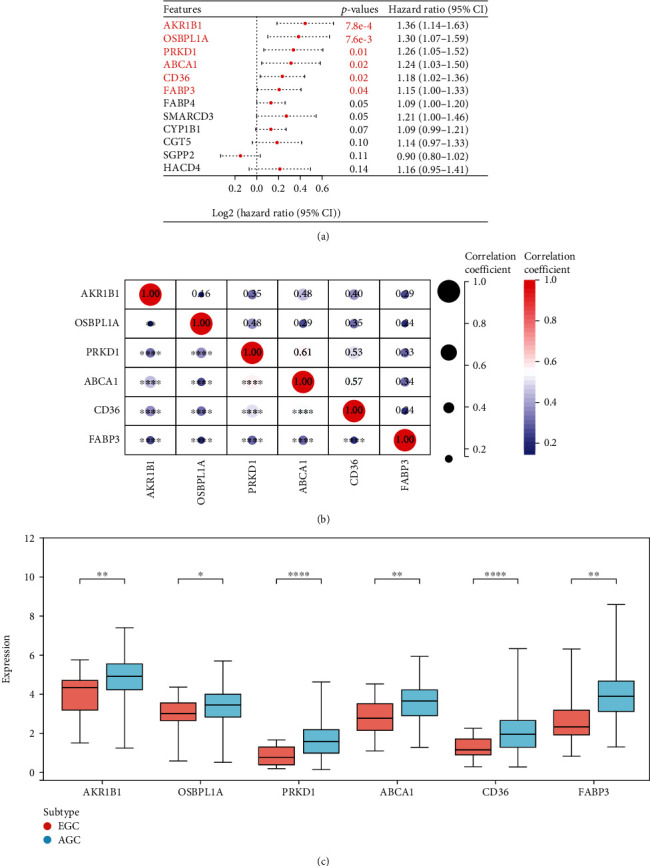
Screening of prognostic lipid metabolism differential genes. (a) Univariate COX analysis was performed on 40 lipid metabolism-related differential genes, and 6 genes were associated with prognosis. (b) Expression levels of six prognostic genes in EGC and AGC. (c) Correlation analysis of 6 prognostic genes.

We conducted univariate Cox regression analysis on the 40 identified differential genes related to lipid metabolism and identified six LMRGs associated with prognosis (Figure 2(a)). Notably, AKR1B1, OSBPL1A, PRKD1, ABCA1, CD36, and FABP3, all upregulated in AGCs, were identified as high-risk prognostic genes (Figure 2(b)). Additionally, a positive correlation was evident among LMRGs in AGC samples at the mRNA level, confirmed by the Pearson correlation test (Figure 2(c)).

### 3.3. Establishment of Molecular Subtypes through Coherent Clustering

**Figure 3:**
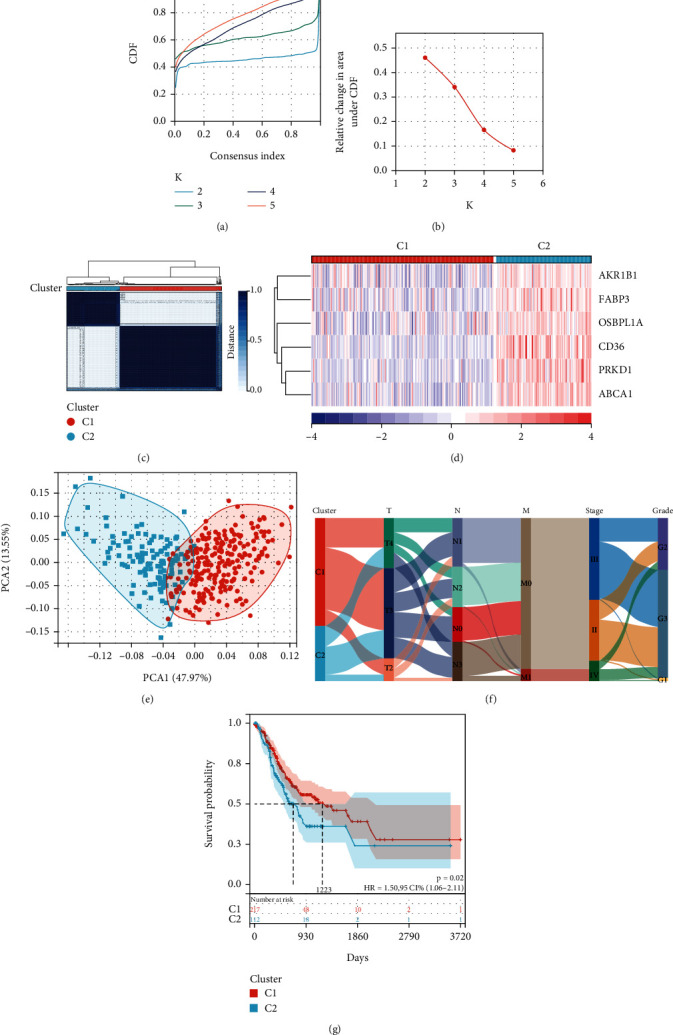
Molecular subtypes constructed by consistent clustering. (a, b) The nearest *k* = 2 is obtained from the cumulative distribution curve and the area under the curve. (c) AGC samples were divided into C1 and C2 subtypes. (d) Heat maps of six different subtypes of prognostic genes. (e) PCA dimension reduction was performed on samples of C1 and C2 subtypes to show differences between the two subtypes. (f) Relationship between different subtypes and clinical features. (g) Survival analysis of C1 and C2 revealed that the C2 subtype had a poorer prognosis than the C1 subtype.

Consistent clustering is an unsupervised clustering method commonly employed for the classification of cancer subtypes. By utilizing the *k*-means algorithm and analyzing the cumulative distribution curve and the area under the distribution curve (Figures 3(a) and 3(b)), we determined that the optimal clustering was achieved when *k* = 2. Subsequently, two clustering schemes were identified, assigning 228 AGC samples to subtype 1 (cluster 1, C1) and 119 AGC samples to subtype 2 (cluster 2, C2) (Figure 3(c)). The heat map showed that lipid metabolism-related genes had a higher expression in group C2 than in group C1 (Figure 3(d)). The PCA plots indicated significant differences between the samples of the two subtypes after dimensionality reduction (Figure 3(e)). Further investigation of the relationship between these two cluster subtypes and various clinical features revealed that patients in group C2 had lower degrees of histological differentiation and higher stages than those in group C1 (Figure 3(f)). Additionally, patients in group C2 exhibited shorter OS than those in group C1 (Figure 3(g)). These findings suggest that LMRGs may influence the development of AGCs through some underlying mechanisms.

### 3.4. Relationship between Molecular Subtypes and Immune Microenvironment

**Figure 4:**
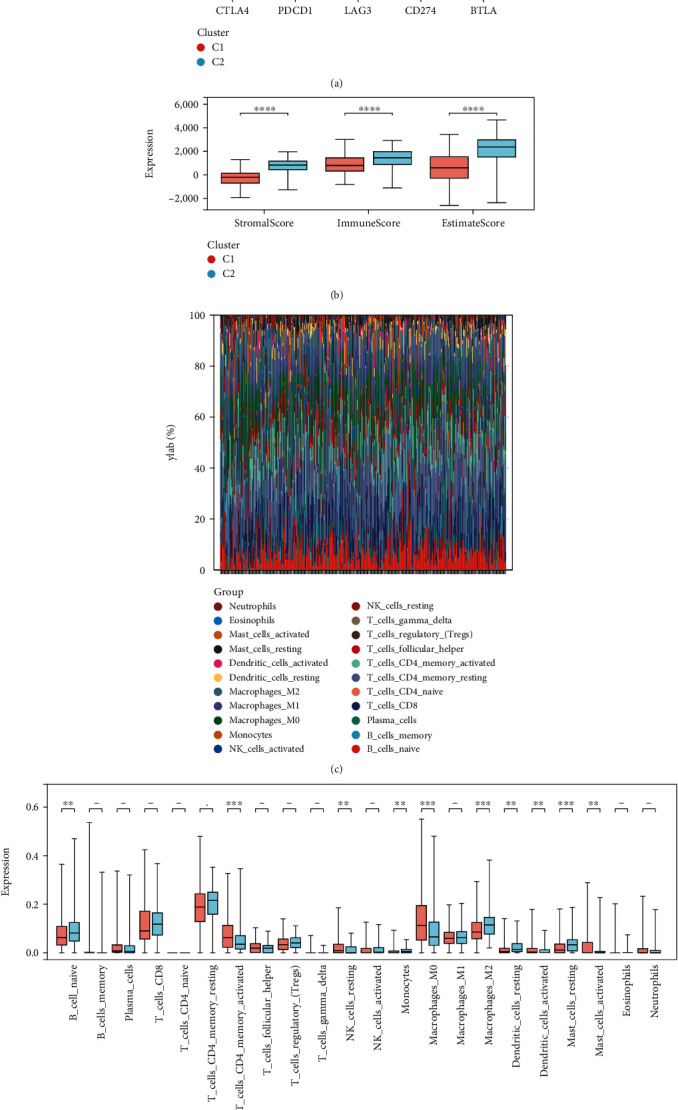
Relationship between molecular subtypes and tumor immune microenvironment. (a) Differences in 5 immune checkpoints of different subtypes. (b) The “estimate” algorithm was used to calculate the purity of different subtypes of tumors. (c) CIBERSORT algorithm was used to calculate the immune cell infiltration of each sample in AGC. (d) CIBERSORT algorithm was used to calculate the difference of immune cell infiltration among different subtypes.

To investigate the association between lipid metabolism and the TIME, we analyzed the composition of the two molecular subtypes in the TME. Our results indicated that the expression levels of immune checkpoints, notably CTLA4, PDCD1, and BTLA, were higher in the C2 group, while LAG3 and CD274 showed no significant differences between C1 and C2 groups (Figure 4(a)). Moreover, we employed the “Estimate” algorithm to evaluate tumor purity between the two subtypes. Compared with C1, C2 had higher matrix scores and immune scores, indicating lower tumor purity (Figure 4(b)). To further explore the reasons for the high immune score in group C2 and the impact of immune cell infiltration on AGC patients, we used the “CIBERSORT” algorithm to estimate the degree of immune cell infiltration. Our analysis indicated that the infiltration degree and average content of various immune cells varied between subtypes. In AGC samples, the “CIBERSORT” algorithm suggested that the average contents of CD4-T cells, M0 macrophages, and CD8-T cells in resting memory were the highest, whereas the average contents of naive CD4-T cells, *γδ* T cells, and eosinophils were the lowest (Figure 4(c)). Furthermore, the types of cells with higher infiltration degree in the C2 group were different. Juvenile B cells, monocytes, M2 macrophages, resting dendritic cells, and resting mast cells were more infiltrated in the C2 group. The infiltration degree of plasma cells, activated memory CD4-T cells, resting natural killer cells, M0 macrophages, activated dendritic cells, and activated mast cells was higher in group C1 (Figure 4(d)). Collectively, these findings suggest the existence of two distinct immunophenotypes in AGCs, which could contribute to a better understanding of the pathogenesis of AGCs and guide the development of treatment regimens.

### 3.5. Establishment of Weighted Gene Coexpression Network

**Figure 5:**
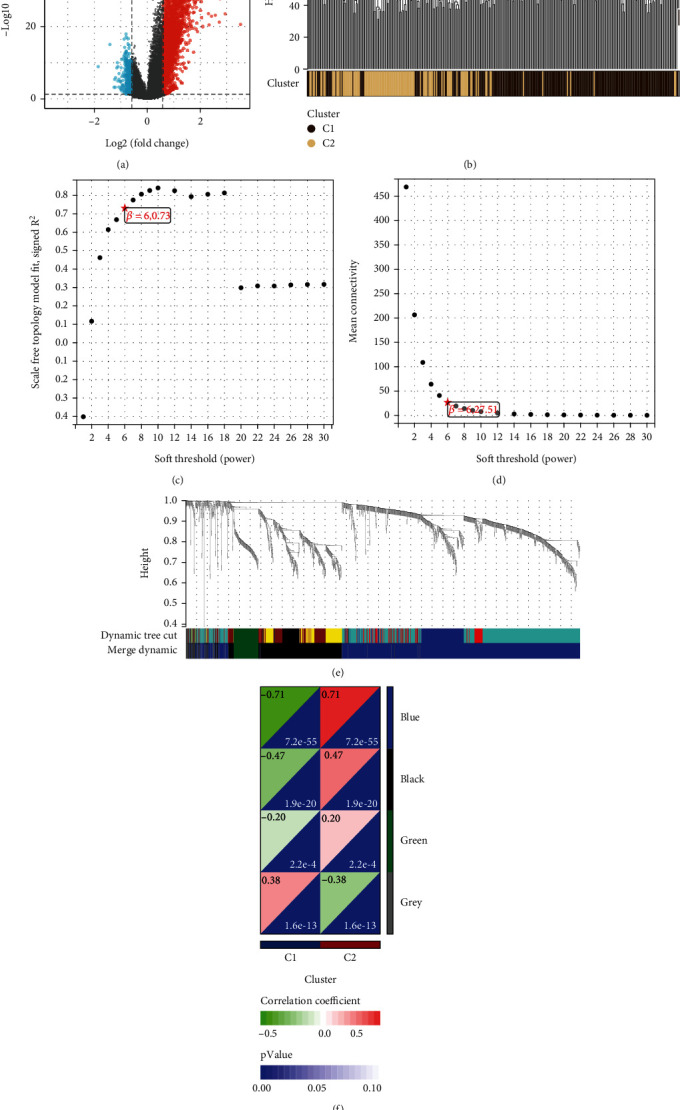
Establishment of weighted gene coexpression network. (a) Differential genes between C2 and C1 were analyzed in the “limma” package, with red representing upregulated genes and blue representing downregulated genes. (b) Clustering of samples from different subtypes of C1 and C2. (c, d) According to scale independence and mean connectivity, the soft threshold is determined to be 6, *R*^2^ is 0.73, and scale-free network is constructed. (e) After the elimination of the first 50% of the smallest MAD genes, they were combined into different modules according to the coexpression relationship of the genes. (f) Except gray, the remaining three modules are correlated with the subtype, and the blue module has the greatest correlation.

To investigate the prognostic differences between the two molecular subtypes in gastric adenocarcinoma, we employed the limma software package to analyze the differences between C1 and C2 subtypes (Figure 5(a)). We set strict screening conditions, requiring a difference multiple of 1.5 times or more, and P.adj to be less than 0.05, ultimately identifying 3022 DEGs. Before conducting the WGCNA, we preprocessed the gene expression data by calculating the MAD for each gene and excluding the top 50% of genes with the smallest MAD. We then removed outlier genes and samples using the “goodSamplesGenes” method of the R software package WGCNA (Figure 5(b)) to ensure reliable results. Subsequently, we constructed scale-free networks using WGCNA. Based on scale independence and mean connectivity (Figures 5(c) and 5(d)), we selected a soft threshold of 6, *R*^2^ greater than 0.73, and module merging threshold of 0.25. We identified three modules, excluding the gray module, for further research. We then analyzed the modules after clustering tree merger (Figure 5(e)) and calculated the correlation coefficient between the module eigenvalue (ME) and clinical phenotypes to study the association between modules and molecular subtypes. We found that the blue module was strongly correlated with lipid metabolism subtypes (*p* = 7.2∗10^−55^, *R* = 0.71), indicating that this module genes are closely related to the prognosis of the AGC molecular subtype (Figure 5(f)).

### 3.6. PPI Network and Enrichment Analysis of Lipid Metabolism-Related Coexpression Modules

**Figure 6:**
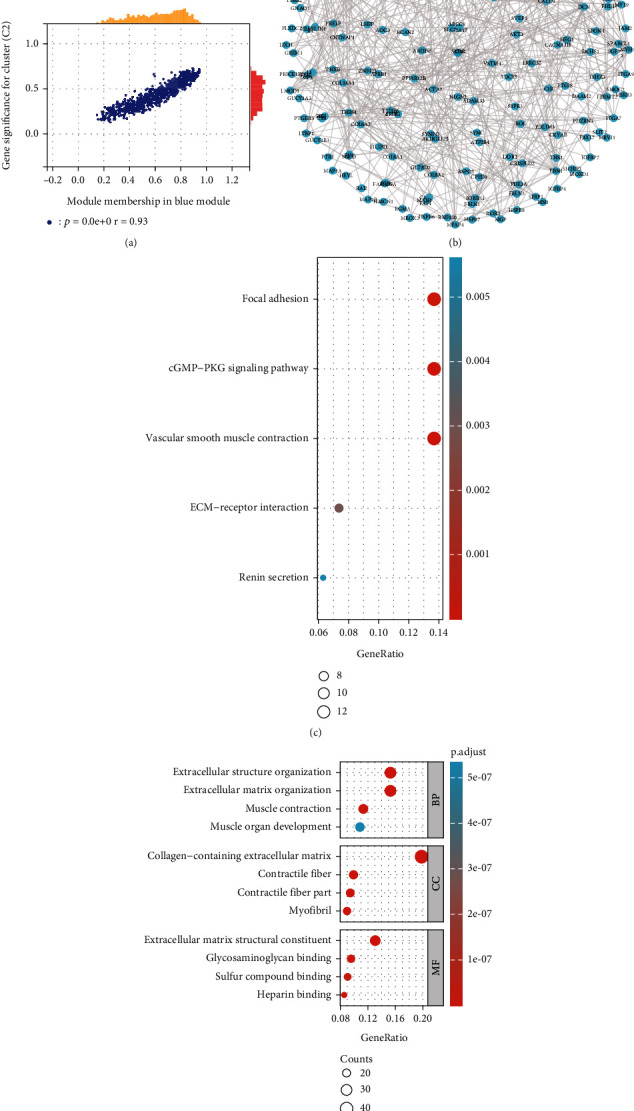
PPI network and enrichment analysis of lipid metabolism-related coexpression modules. (a) Scatter plots of interactions between module membership and gene significance of genes in the blue module. (b) PPI network interaction map constructed by hub gene in blue module. (c, d) Hub gene was used for GO/KEGG enrichment analysis.

Following the WGCNA analysis, we identified the blue module consisting of 991 genes that showed high correlation with the molecular subtypes (Figure 6(a)). Subsequently, employing stringent criteria, we designated 220 genes with significant connectivity as hub genes and conducted a protein-protein interaction (PPI) network analysis utilizing the STRING database. Visualization of this network was facilitated using Cytoscape 3.6.1 software (Figure 6(b)). To decode the biological relevance of these hub genes, we further conducted Gene Ontology (GO) and KEGG enrichment analysis of these hub genes to elucidate their biological significance. GO enrichment analysis unveiled significant associations with biological processes encompassing extracellular matrix organization, muscle contraction, and muscle tissue development. Concurrently, cellular component (CC) analysis highlighted enrichment in extracellular matrix components, including collagen and contractile fibers, while molecular function (MF) analysis underscored roles in extracellular matrix structural constituents, heparin binding, and glycosaminoglycan binding. Complementing these findings, KEGG pathway analysis delineated enrichment in pivotal pathways such as cGMP-PKG signaling, vascular smooth muscle contraction, renin secretion modulation, extracellular matrix- (ECM-) receptor interactions, and adhesive plaques (Figures 6(c) and 6(d)). Collectively, these insights shed light on potential mechanistic underpinnings driving disparate prognostic outcomes observed between the C2 and C1 cohorts.

### 3.7. AGC risk characteristics were developed and verified, and a line map was established by GSEA

**Figure 7:**
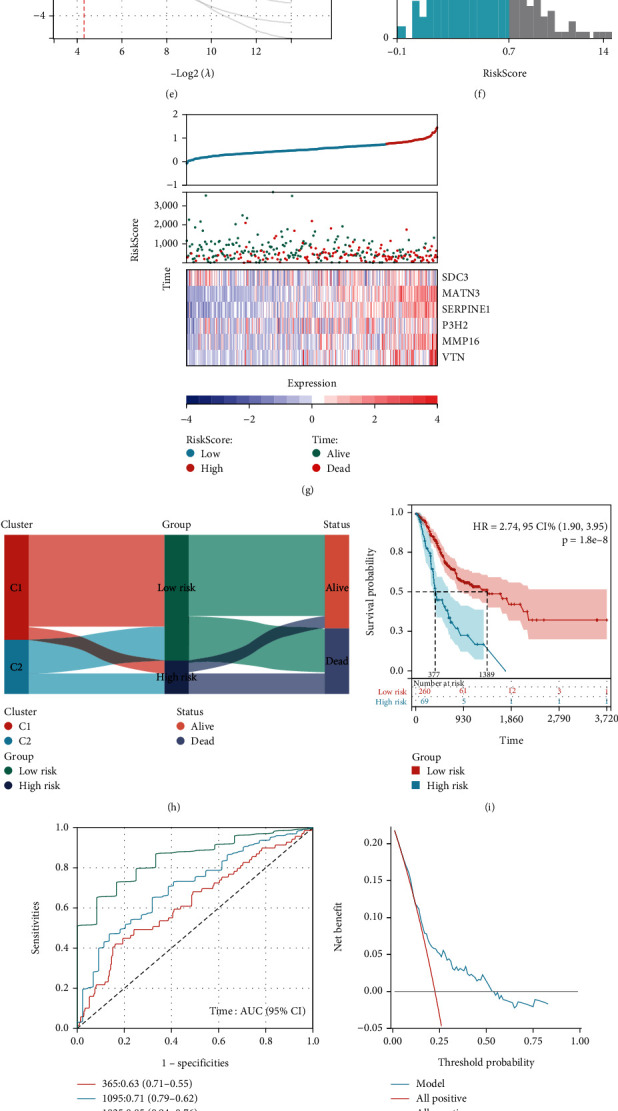
AGC risk characteristics were developed and verified, and a line graph was established using GSEA. (a) Five pathways activated in C2 were selected by GSEA based on differential genes between C2 and C1. (b, c) The batch effect was removed by combining the AGC samples of TCGA-STAD and GSE62254. (d, e) LASSO-Cox regression was used to construct a prognostic model. When *λ* = 0.05, a prognostic model containing 6 genes was obtained. (f) X-Tile software was used to calculate the optimal cut-off value of 0.74. (g) Prognostic heat maps showed the survival status, risk score, and distribution of 6 gene expressions of AGCs in high-risk and low-risk groups. (i–k) Using TCGA-STAD as the training set, KM curve, ROC curve, and DCA curve were used to determine the prognostic value of the model. (l–n) GSE62254 was used as external validation to verify the prognostic value of the model by KM curve, ROC curve, and DCA curve. (o, p) Based on TCGA-STAD, risk score combined with TNM stage, age, gender, differentiation degree, and stage to draw calibration curve. (q) ROC curve was plotted to predict the prognosis of AGC patients.

To elucidate the risk characteristics of AGC, we developed and validated a robust model, subsequently generating a schematic through GSEA. Employing GSEA to discern differential biological processes and pathways between C1 and C2 samples, notable activations were observed in pathways such as vascular smooth muscle contraction, cell adhesion molecules, and extracellular matrix interactions predominantly in the C2 cohort, along with heightened lipid metabolism in AGCs (Figure 7(a)). Merging TCGA-STAD training set with the GSE62254 AGC verification samples after batch effect correction (Figures 7(b) and 7(c)), we pinpointed extracellular stromal genes of core enrichment. Utilizing the LASSO-Cox method, our prognostic AGC model crystallized around six pivotal genes (Figures 7(d) and 7(e)). RiskScore = 0.122 × SCD3 + 0.145 × MATN3 + 0.120 × SERPINE1 + 0.034 × P3H2 + 0.061 × MMP16 + 0.059 × VTN. Through rigorous validation and multivariate Cox analysis, this model emerged as an autonomous risk determinant for AGC (Table 2). The X-Tile software calculated an optimal cut-off value of 0.74 (Figure 7(f)). We divided AGC patients in TCGA cohort into two risk subgroups based on the optimal risk score cut-off value. The survival heat map demonstrated the patient's survival rate based on the risk score and showed the differential expression of six genes between the two groups (Figure 7(g)). The mulberry chart illustrated the relationship between lipid metabolic subtypes, risk scores, and survival status (Figure 7(h)). OS was lower in the high-risk group than in the low-risk group (Figure 7(i)). The time-dependent ROC curve verified the accuracy of the risk curve at 1, 3, and 5 years, with areas under the curve of 0.63, 0.71, and 0.85, respectively (Figure 7(j)). The DCA curve also demonstrated the clinical practicability of the model (Figure 7(k)). These results were validated in the external dataset GSE62254, where the samples meeting the inclusion criteria were divided into high- and low-risk groups based on a cut-off value of 0.74. KM survival analysis showed that overall survival was significantly lower in the high-risk group than in the low-risk group. In the validation cohort, the AUC values predicting 1-, 3-, and 5-year survival were 0.70, 0.61, and 0.60, respectively.

To facilitate clinical use, we constructed a column graph combining the risk score with TNM stage, age, sex, degree of differentiation, and stage and plotted a calibration curve based on TCGA-STAD dataset. ROC curves show that our columns have good accuracy in predicting 1-, 3-, and 5-year survival rates (Figures 7(o)–7(q)). In conclusion, these models accurately distinguish the high- and low-risk groups of patients with AGC and have a high predictive value in predicting 1–5-year survival of patients with AGC.

### 3.8. Drug Sensitivity Analysis

**Figure 8:**
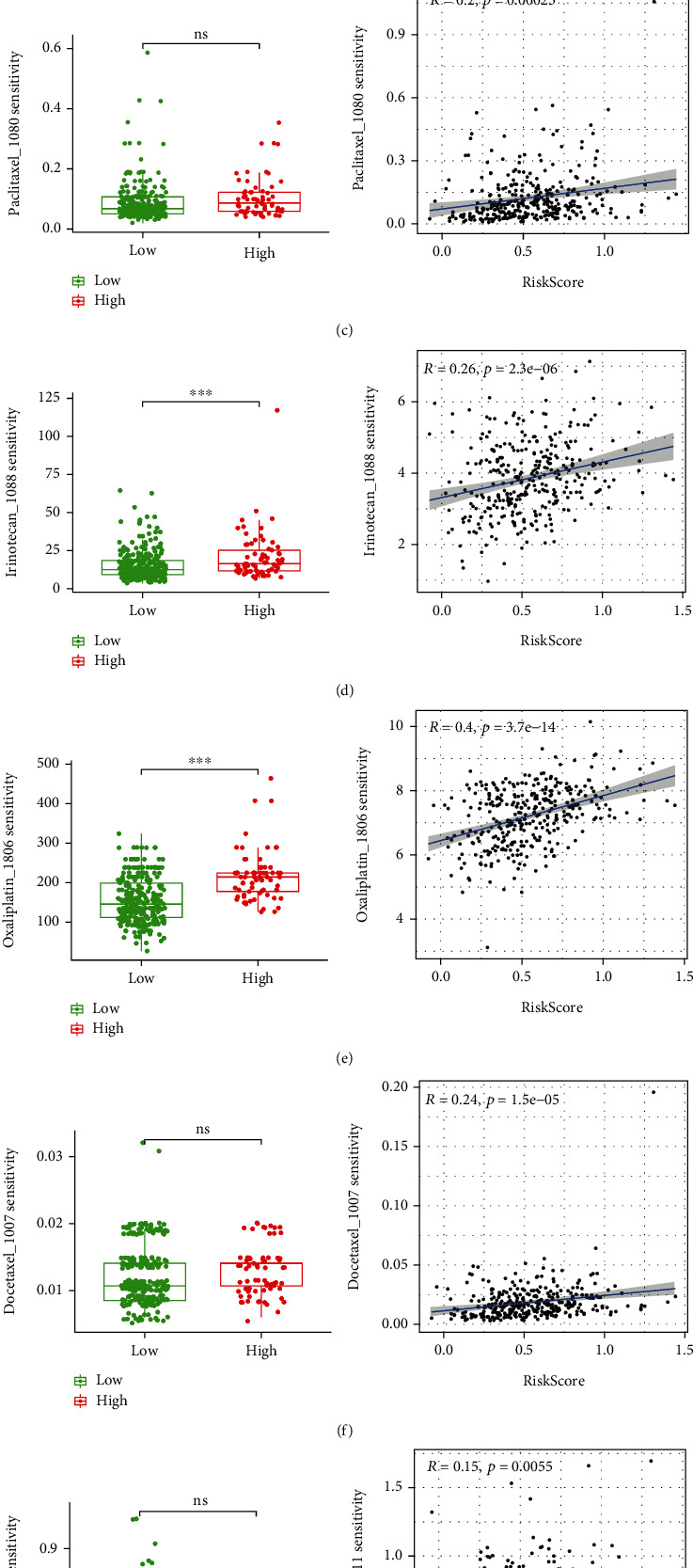
Analysis of drug sensitivity. Comparison of projected IC_50_ values and correlation of risk scores with projected IC_50_ values between high-risk and low-risk groups of (a) 5-fluorouracil, (b) cisplatin, (c) paclitaxel, (d) irinotecan, (e) oxaliplatin, (f) docetaxel, (g) epirubicin, and (h) camptothecin.

Given the therapeutic significance of chemotherapy in AGC management, we harnessed the “oncoPredict” R package to delineate the interplay between drug sensitivity and our risk scores. Evaluating prevalent chemotherapy agents for gastric cancer, our findings spotlighted differential sensitivities across risk groups. Our results showed that the low-risk group had higher sensitivity to 5-fluorouracil, cisplatin, irinotecan, oxaliplatin, and camptothecin compared to the high-risk group. Moreover, as risk score increased, the IC_50_ values mirrored this trend. Although the sensitivities of paclitaxel, docetaxel, and epirubicin did not show significant differences between high-risk and low-risk groups, their sensitivities were negatively correlated with risk scores (Figures 8(a)–8(h)). Collectively, our findings indicate that our risk model can facilitate clinical decision-making for the treatment of AGC patients.

### 3.9. The expression and role of lipid metabolism-related prognostic genes in STAD were examined using single-cell RNA-seq analysis

**Figure 9:**
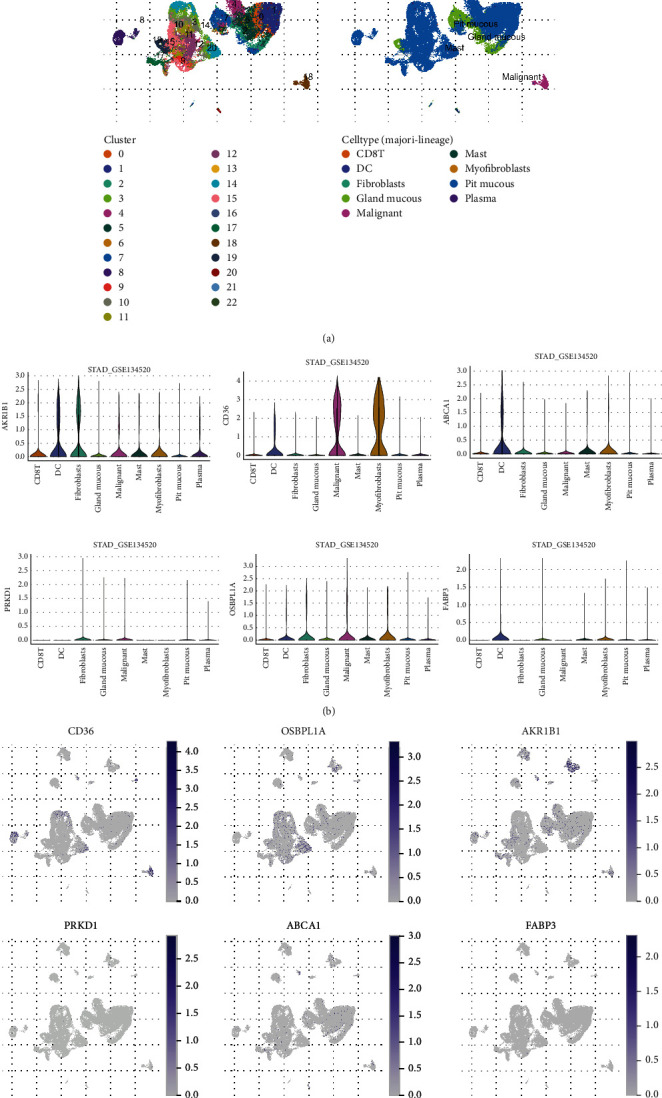
Validation of the expression and role of lipid metabolism-related prognostic genes in STAD by single-cell RNA-seq. (a) In the GSE134520 dataset, 23 cell clusters were obtained after dimension reduction, and each cell type was identified by marker genes. (b) Violin plots of the expression profiles of 6 differential prognostic genes of lipid metabolism in different cells. (c) Distribution of 6 different lipid metabolism differential prognostic genes in different cells.

**Figure 10:**
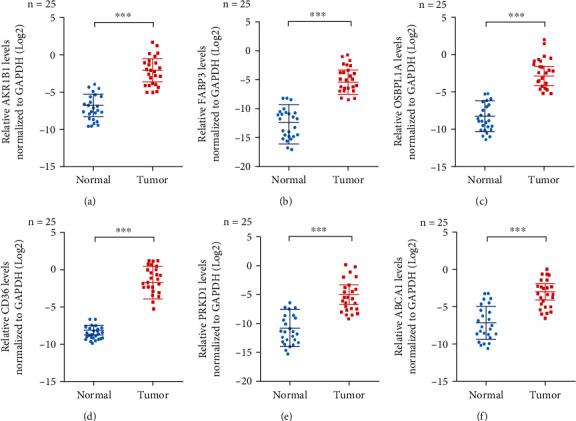
The 6 LMRG expressions in gastric cancer tissue. qRT-PCR results showed that six LMRG genes were differentially expressed between cancer and paracancerous tissues. (a) AKR1B1, (b) FABP3, (c) OSBPL1A, (d) CD36, (e) PRKD1, and (f) ABCA1 were highly expressed in cancer tissues. The data are the mean ± SD of three independent experiments. ^∗∗^*p* < 0.01; ^∗∗∗^*p* < 0.001.

To identify the specific cell types expressing these genes in the STAD tumor microenvironment, we analyzed GSE134520 data and identified nine distinct clusters after cell annotation (Figure 9(a)). Among the clusters, we found that six lipid metabolism-related prognostic genes were differentially expressed across different cells (Figures 9(b) and 9(c)). The AKR1B1 gene was predominantly expressed in fibroblasts, dendritic cells, and CD8-T cells, while the CD36 gene was mainly expressed in malignant cells, myoblasts, and dendritic cells. The ABCA1 gene was primarily expressed in dendritic cells, myoblasts, and mast cells, whereas the PRKD1 gene was predominantly expressed in fibroblasts. The OSBPL1A gene showed high expression in malignant cells, myoblasts, and fibroblasts, while the FABP3 gene was mainly expressed in dendritic cells. Our RNA-seq data verified the differential expression of these genes in different cell types and provided further evidence of their potential involvement in STAD carcinogenesis. To further elucidate the expression of AKR1B1, FABP3, OSBPL1A, CD36, PRKD1, and ABCA1 in gastric cancer, we first detect their expression in the human GC tissues and paired normal tissues, and the results showed that the mRNA levels of these six LMRGs were significantly higher in tumor tissues compared with those in normal tissues by qRT-PCR (Figures 10(a)–10(e)).

## 4. Discussion

GC remains a significant health problem with high incidence and poor prognosis, especially for advanced GC. Despite new advances in GC treatment, the 5-year survival rate in China remains low due to the heterogeneity and complex etiology of GC [29]. Biomarkers currently used to monitor the prognosis of advanced GC, such as CEA, CA724, CA199, CA125, and pepsinogen I/II, have limitations such as poor sensitivity and specificity. It is also challenging to predict the efficacy of immunotherapy [30]. Therefore, there is a pressing need to establish an effective and relatively accurate prognostic parameter and risk classification method.

Recent studies have shown that metabolic reprogramming plays a crucial role in the occurrence and development of GC. Lipid metabolism, in addition to the well-known Warburg effect, has also been shown to be essential [31]. Lipids, including fatty acids, phospholipids, and cholesterol, can serve as an important energy source for cancer cells, an essential component of the cell membrane, and also cytokines to transmit signals [32]. Lipid metabolism disorders occur in the entire process of GC, including precancerous lesions to advanced GC. A variety of lipid abnormalities are also vital risk factors for GC [10].

Lipid metabolism comprises lipid uptake, synthesis, and oxidative decomposition, and changes in lipid biology are involved in the invasion, metastasis, and death of malignant tumors [33, 34]. For instance, CPT1A, a key enzyme of fatty acid oxidation (FAO), promotes lymph node metastasis of GC [35], and apolipoprotein C2 (Apo2) plays a significant role in peritoneal metastasis of GC [36]. Ferroptosis, a recently discovered form of regulated cell death, is inseparable from lipid peroxidation [37]. Previous studies suggested that iron death resistance is one of the causes of drug resistance in advanced GC [38–40].

Our investigation unveiled distinct expression patterns of LMRGs in AGC compared to EGC tissues, and these patterns exhibited a close correlation with immune cell infiltration. Among the identified LMRGs, six were associated with prognosis, enabling the construction of two molecular subtypes based on their expression levels. Subtype C2, characterized by high LMRG expression, exhibited a poorer prognosis compared to subtype C1, characterized by low LMRG expression. Aldo-keto reductases (AKRs), a significant enzyme class present across various organisms, including humans, play diverse biological roles encompassing metabolism, lipid synthesis, and DNA repair. Within this family, AKR1B1 has been implicated in gastric cancer progression, regulated by the binding of ZNF521 and EB1, promoting proliferation, migration, and invasion of gastric cancer cells [41]. AKR1BA induces cellular senescence in subcutaneous adipose tissues, modulating cellular aging through activation of the PI3K/Akt and p38 MAPK pathways [42]. OSBPL1A is a molecule that mediates cholesterol metabolism. Current research on OSBPL1A is limited, but existing studies suggest that it influences the production of HDL-C in the liver and intestines in conjunction with ABCA1 and also acts as a crucial prognostic factor in colorectal cancer [43, 44]. The PRKD1 gene encodes a protein known as protein kinase D1 (PRKD1), which belongs to a significant class of signal transduction protein kinases. It can modulate a multitude of cellular biological processes, such as proliferation, apoptosis, cell polarity, cell-cell interaction, extracellular matrix adhesion, and cell cycle control, through phosphorylation. PRKD1 profoundly affects the gene expression in brown adipose tissue (BAT) and its differentiation. Knockout of PRKD1 markedly reduced the expression of Myf5 and MyoD genes in BAT [45]. FABP3 is a member of the fatty acid-binding protein (FABP) family, also known as the cardiac-type FABP. It is a small molecular weight protein consisting of a 15 kDa polypeptide chain, functioning both in the cytoplasm and on the cell membrane [46]. FABP3 is predominantly expressed in the heart and skeletal muscles. It regulates lipid metabolism by binding and transporting free fatty acids (FFA), facilitating the synthesis and *β*-oxidation of triacylglycerol (TAG) [47, 48]. FABP3's function is also associated with inflammatory conditions, insulin sensitivity, and metabolic syndrome. Overexpression of FABP3 has been linked to obesity, type 2 diabetes, and cardiovascular diseases [49]. Hence, FABP3 presents itself as a significant molecular target for regulating lipid metabolism and metabolic diseases. CD36 is a transmembrane protein extensively expressed in various cell types, with its encoding gene located on human chromosome 7q11.2. As a multifunctional protein, CD36 participates in several physiological processes, including lipid metabolism, immune responses, cell adhesion, and signal transduction. Its expression levels on the cell membrane are intimately associated with the onset and progression of numerous diseases such as cardiovascular diseases, metabolic syndrome, and obesity [50]. In recent years, increasing research indicates the significant role of CD36 in tumors. It performs vital functions in tumorous cell lipid metabolism, metastasis, and immune evasion [51]. Studies have revealed that overexpression of CD36 is commonly observed in various tumors and can regulate lipid uptake, oxidation, and synthesis, thereby influencing the growth and migration of cancer cells [8]. The metastasis of tumors is an essential factor affecting the prognosis of patients and may be a crucial reason for the shorter overall survival of C2. The expression levels of these six genes can reflect the prognosis of AGC patients to some extent, and patients can be divided into high-risk and low-risk cohorts. Herein, using single-cell RNA-seq, we analyzed lipid metabolism-related genes in STAD, identifying differential expressions across cell types. Notably, AKR1B1, CD36, ABCA1, PRKD1, OSBPL1A, and FABP3 showed varied expressions in specific cells, suggesting their significant roles in STAD carcinogenesis, further confirmed by elevated mRNA levels in tumor tissues via qRT-PCR. Together, this risk feature shows high predictive value in terms of overall survival and may serve as an independent prognostic indicator for patients with AGC.

To further clarify the internal association and difference of the two newly discovered molecular subgroups, WGCNA was performed according to C1 and C2 differential genes. In the module most related to lipid metabolism phenotype, we did enrichment analysis according to hub genes and constructed PPI network. Cell adhesion, biological adhesion, extracellular matrix, structural molecule activity, cGMP PKG signaling pathway, vascular smooth muscle contraction, vascular smooth muscle contraction, focal adhesion, and other functions were enriched. This may suggest an important reason that affects the prognosis of both [52]. Then, we constructed a GSEA according to the differential genes of the two groups, in which the extracellular matrix pathway was activated in C2 relative to C1, so we constructed a risk model based on six extracellular matrices, including SDC3, MATN3, SERPINE1, P3H2, MMP16, and VTN. This has significant prognostic value. Consistent results were also obtained using data from the GEO database. We can divide AGC into high-risk and low-risk groups according to risk score. The DCA curve further helps us to obtain better clinical static benefits. In addition, we constructed nomogram by combining risk score with TNM stage, grade, stage, gender, and age of tumor. In addition, the calibration chart shows that according to our nomogram, the predicted 1-, 3-, and 5-year survival rates are close to the actual situation. Therefore, the prognostic characteristics of LMRGs can accurately predict the survival outcome of AGC patients, enabling clinicians to easily estimate the outcome and make individual prognosis and treatment decisions.

Both GO/KEGG and GSEA seem to suggest that our extracellular matrix plays an important role in the difference between the two clusters. Tumor extracellular matrix is composed of collagen, elastin, proteoglycans, and glycoproteins and is used for the complex, interconnected macromolecular network surrounding and supporting cells in organs and tissues [53]. For example, fibroblasts (CAFs) in the extracellular matrix can promote the invasion and metastasis of GC [54]. Extracellular matrix fibrillin 1 (FBN1) can also promote the development of GC through succinylation [55]. In conclusion, extracellular matrix can be used not only as a regulator of tumor therapy but also as a target of tumor therapy and also has important value for diagnosis and prognosis [53].

As previously mentioned, in addition to the extracellular matrix, immune cells also belong to an important part of the tumor microenvironment. The tumor immune microenvironment is closely related to the clinical characteristics and prognosis of GC [56]. In this study, we used the estimate algorithm to score the tumor purity. We found that C2 with poor prognosis had higher stromal score and immune score than C1. This proves that its tumor purity is low and also suggests that AGC with high expression of lipid metabolism genes is more immunogenic. At the same time, the analysis results of immune checkpoints also tell us that C2 has higher expression than C1, which provides a possible basis for immunotherapy. Studies have suggested that after PD-L1 blockade, FABPs can ingest more fatty acids, thereby prolonging the life span of tissue resident memory cells [57]. CIBERSORT also suggested that immune cell infiltration was significantly different between the two groups. Suggesting a link between our lipid metabolism and immunity. In fact, the relationship between lipid metabolism and tumor immunity is complex and contradictory. For example, tissue resident memory cells absorb fatty acids from the TME through CD36 and FABPs to produce antitumor cytokines. Conversely, high-dose fatty acids also cause effector T cell exhaustion or stimulate PPAR-*β* in regulatory T cells and fatty acid oxidation to mediate the immunosuppressive response [58]. PD-1 inhibitors potentiated the antitumor effect of CD36 blockade [59]. This proves once again that lipid metabolism can be used as the target of antitumor.

In this study, we investigated the impact of lipid metabolism on AGC and identified two distinct molecular subtypes through consensus clustering. These subtypes were able to predict prognosis and immune status based on the expression of lipid metabolism genes. Furthermore, we explored the biological mechanisms underlying the differences between the two subtypes, analyzed the relationship between time and lipid metabolism, and assessed their impact on the prognosis of AGC. Finally, we developed a nomogram that combines clinical information to accurately predict the prognosis of patients with AGC.

In conclusion, our findings demonstrate that LMRG subtypes are associated with prognosis and immune microenvironment in patients with AGC. By utilizing bioinformatic methods, we identified key genes in the network of lipid metabolism pathways and confirmed the prognostic significance and immunogenomic importance of LMRGs in AGC. Our LMRG model may serve as a powerful tool for predicting survival and guiding treatment in patients with AGC.

## Figures and Tables

**Table 1:** Primer sequences of six LMGRs.

Primer	Sequence
AKR1B1-forward	TTTTCCCATTGGATGAGTCGG
AKR1B1-reverse	CCTGGAGATGGTTGAAGTTGG
OSBPL1A-forward	TCCGAAGAAAAAGACTGTGGTG
OSBPL1A-reverse	CAGTTAGGCGCTGTAGGAAGC
PRKD1-forward	TTCTCCCACCTCAGGTCATC
PRKD1-reverse	TGCCAGAGCACATAACGAAG
ABCA1-forward	GGTGTTGAAAGTCTCGAACATG
ABCA1-reverse	GGGAAAACCCACCATACCTAA
CD36-forward	CTTTGGCTTAATGAGACTGGGAC
CD36-reverse	GCAACAAACATCACCACACCA
FABP3-forward	TTCTGGAAGCTAGTGGACAG
FABP3-reverse	TGATGGTAGTAGGCTTGGTCAT
GAPDH-forward	GTGAAGGTCGGAGTCAAC
GAPDH-reverse	GTTGAGGTCAATGAAGGG

**Table 2:** Univariate and multivariate Cox regression analyses of clinical characteristics and risk scores in AGC.

Characteristics	Total (*N*)	Univariate analysis		Multivariate analysis	
		Hazard ratio (95% CI)	*p* value	Hazard ratio (95% CI)	*p* value
Age	291	1.022 (1.004-1.040)	0.018	1.030 (1.011-1.050)	0.002
T	291				
T2	61	Reference			
T3	148	1.234 (0.756-2.013)	0.400		
T4	82	1.356 (0.792-2.320)	0.267		
N	291				
N0	83	Reference			
N1	79	1.262 (0.754-2.112)	0.376	1.021 (0.536-1.946)	0.949
N2	66	1.311 (0.761-2.258)	0.330	1.071 (0.488-2.353)	0.864
N3	63	2.098 (1.265-3.479)	0.004	1.265 (0.572-2.796)	0.561
M	291				
M0	271	Reference			
M1	20	1.610 (0.841-3.082)	0.150		
Sex	291				
Female	110	Reference			
Male	181	1.376 (0.934-2.027)	0.106		
Grade	291				
G1	6	Reference			
G2	94	2.268 (0.312-16.507)	0.419		
G3	191	2.558 (0.356-18.404)	0.351		
Stage	291				
Stage 1	26	Reference			
Stage 2	99	1.264 (0.580-2.755)	0.556	1.509 (0.647-3.518)	0.341
Stage 3	134	1.637 (0.779-3.441)	0.193	1.658 (0.599-4.591)	0.330
Stage 4	32	2.831 (1.245-6.437)	0.013	3.253 (1.114-9.499)	0.031
Risk score	291	5.982 (3.232-11.075)	<0.001	5.496 (2.903-0.406)	<0.001

## Data Availability

The data could be download at (https://portal.gdc.cancer.gov/, https://xenabrowser.net/, and https://www.ncbi.nlm.nih.gov/geo/query/acc.cgi?acc=GSE62254) and the code used during the current study are available from the corresponding author on reasonable request.
